# A Multimodal Agentic AI Framework for Intuitive Human–Robot Collaboration

**DOI:** 10.3390/s26061958

**Published:** 2026-03-20

**Authors:** Xiaoyun Liang, Jiannan Cai

**Affiliations:** School of Civil & Environmental Engineering, and Construction Management, The University of Texas at San Antonio, San Antonio, TX 78249, USA; xiaoyun.liang@my.utsa.edu

**Keywords:** human–robot collaboration, multimodal interaction, timber assembly

## Abstract

Widespread acceptance of collaborative robots in human-involved scenarios requires accessible and intuitive interfaces for lay workers and non-expert users. Existing interfaces often rely on users to plan and issue low-level commands, necessitating extensive knowledge of robot control. This study proposes a multimodal agentic AI framework integrating natural user interfaces (NUIs) to foster effortless human-like partnerships in human–robot collaboration (HRC), which enhance intuitiveness and operational efficiency. First, it allows users to instruct robots using plain language verbally, coupled with gaze, revealing objects precisely. Second, it offloads users’ workload for robot motion planning by understanding context and reasoning task decomposition. Third, coordinating with AI agents built on large language models (LLMs), the system interprets users’ requests effectively and provides feedback to establish transparent communication. This proof-of-concept study included experiments to demonstrate a practical implementation of the agentic AI framework on a mobile manipulation robot in the collaborative task of human–robot wood assembly. Seven participants were recruited to interact with this AI-integrated agentic robotic system. Task performance and user experience metrics were measured in terms of completion time, intervention rate, NASA TLX survey for workload, and valuable insights of practical applications were summarized through a qualitative analysis. This study highlights the potential of NUIs and agentic AI-embodied robots to overcome existing HRC barriers and contributes to improving HRC intuitiveness and efficiency.

## 1. Introduction

Alongside the great advancement of artificial intelligence (AI), robotic systems are extending far beyond the confined spaces of industrial assembly lines to dynamic, human-centric environments. Robots are increasingly integrated as assistants in our lives, such as social robots, providing companionship and emotional support to children and the elderly [1]; collaborative robots, where “cobots” work alongside human operators [2]; and humanoid robots for household chores [3]. HRC is a promising solution to labor-intensive industries in the background of a shrinking labor market. However, the complexity of the interfaces required to interact with robots hinges on the actual usage of robots in these sectors such as construction [4], healthcare [5], and agriculture [6]. It presents a pressing need for easy and intuitive interaction to minimize the cognitive gap between a user’s goal and the robot’s corresponding actions.

Natural user interfaces (NUIs) leverage everyday human behaviors like speech, gesture, and gaze to establish intuitive interactions [7,8]. The intuitive human–robot interaction makes advanced robotic systems accessible to lay workers. Especially in construction, large language models (LLMs) are leveraged to enhance robot control and scene understanding [9]. However, human language can be ambiguous, which is worse in the unstructured and complex context of construction tasks. Conveying precise spatial information for HRC is essential in these environments. Misinterpreted instructions due to language ambiguity can cause critical errors. These errors may lead to accidents or major project delays. This challenge highlights a pressing need for fusing multiple communication cues to ensure safety and efficiency in construction settings [10].

The integration of agentic AI into multimodal interaction has the potential to offload cognitive work from the human operator to the robotic system [11]. An agentic AI is capable of performing autonomous and goal-oriented actions in dynamic environments with minimal human supervision. It leverages LLMs as core reasoning engines and translates this reasoning into tangible actions [12]. In HRC, an AI agent is applied to translate human intentions to robot actions [13]. This could allow workers, who may hold limited skill sets, to work with a robot without extra burden using NUIs (e.g., speech, gaze). Achieving robust performance in dynamic environments also requires adaptive low-level control strategies for mobile manipulators [14]. To enable autonomous systems in complex sequential tasks, an AI agent is embodied in a robot to decompose users’ instructions for robot manipulation in a simulated world [13]. Few studies have investigated the integration of agentic AI and multimodal interaction for intuitive HRC in real-world applications.

**Figure 1:**
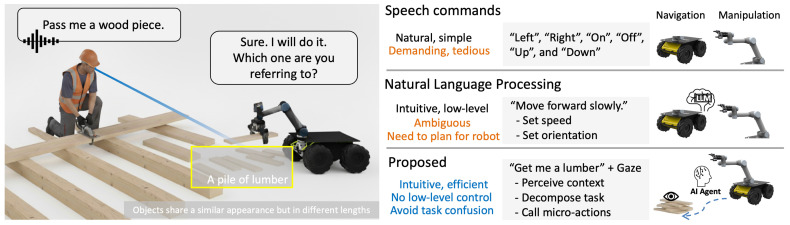
The proposed agentic AI workflow fused with gaze (blue arrow in the scene) and verbal data establishes transparent communication and effortless robot operation in human–robot collaboration (GenAI was used to generate the scene on the left).

To address the challenge of practical multimodal interaction in HRC and enhance intuitive HRC, this study proposes an agentic AI framework integrating gaze and verbal context via LLMs. It coordinates multiple AI agents to capture audio, identify objects indicated by gaze, generate action plans for a robot, provide feedback to users, and translate plans to executable robot commands. Our method incorporates LLMs to compile actions and generate feedback, establishing a transparent communication between humans and robots (Figure 1). The main contributions are summarized below:

- We propose a multimodal agentic AI framework, establish transparent communication via natural user interface (convey intention through gaze), and fuse with the robot system to construct complex temporal controls.
- This framework was implemented in a real-world application—a timber assembly HRC. Our system utilizes multiple AI agents to understand, communicate, and generate executable robot commands, incorporating language and gaze for inference. It provides a clear, practical workflow of an agentic AI application in HRC for further research.
- A dual-focus evaluation of this system was conducted including subjective and objective metrics to gain practical insights into the system’s real-world application. We also compared it against the pure LLM method to demonstrate its feasibility, usability, and acceptability to humans.

## 2. Related Work

### 2.1. Natural User Interfaces (NUIs) for Intuitive HRC

Advances in AI and robotics have resulted in a wide array of assistive and collaborative robots, transforming traditional workflows. Recent innovations in interfaces, such as brain–computer interaction and wearable devices, have enabled more seamless communication and cooperation between humans and robots. For example, Angrisani et al. used non-invasive brain–computer interfaces (BCIs), enabled by an augmented reality headset, to allow an operator to issue direct, hands-free commands to a robot for smart manufacturing [15]. In such cases, advanced interfaces enable intuitive, real-time collaboration between humans and robots, integrating human intent into robotic control without adding cognitive burden and thus unlocking effective cooperation in dynamic environments. These advanced interfaces enable intuitive, real-time collaboration by integrating human intent directly into robotic control without increasing cognitive burden, thereby unlocking the potential for effective HRC in dynamic, real-world settings.

The importance of an advanced interface for seamless HRC is amplified in the construction industry. Construction sites are characterized by rapidly changing environments, the frequent movement of personnel and equipment, and the presence of multiple hazards that contribute to heightened safety risks. Therefore, construction HRC interfaces must be exceptionally natural and intuitive, allowing workers to interact with robots seamlessly while maintaining their focus on safety and complex, dynamic tasks [16,17]. A range of human–robot interfaces [18,19,20] have been designed and tested for HRC in construction. For instance, Liu et al. proposed a BCI to teleoperate a robot by continuously translating workers’ brainwaves into specific commands [21]. Czarnowski et al. tested virtual reality (VR) and audible sound systems to facilitate communication in HRC by presenting explicit visual and sound cues to teach construction workers about the robot’s behavior [22]. However, translating these innovations from the lab to the real-world construction site remains challenging. These advanced systems face practical limits due to their intensive setup and pre-training requirements and high susceptibility to inherent human variability. For example, the intra- and inter-individual variability of brain waves requires frequent recalibration to accurately translate brain waves into actionable commands. This challenge underscores the critical need for HRC to evolve toward intelligent, adaptive robotic mediators that enable intuitive and effortless human-to-human-like collaboration.

### 2.2. Agentic AI in HRC

Agentic AI-empowered systems are emerging as a transformative approach for achieving the intuitive HRC necessary for real-world success. Agentic AI systems are defined by making decisions and performing tasks with limited or no human intervention. These independent systems automatically respond to conditions to produce process results. In HRC, agentic AI serves as a major catalyst for robot implementation. Agentic AI transforms the robot from a passive tool controlled by the interface into a proactive, intelligent partner that can anticipate needs and react to human mental and physical states. Agentic AI eliminates the operation barriers in traditional HRC by restructuring the interaction into a system spanning from audio control (execute robot via natural language) [9], intention-aware AI agent planner (agent reasons and plans for robot) [23,24], to AI-embodied collaborative robot (agent reasons, plans and operates a robot) [25]. Thus, in agentic AI-empowered HRC, communication shifts away from a one-way human-to-robot command system to a sophisticated two-way loop of intent sharing and adaptation.

An important step in achieving an agentic AI-empowered HRC system is to establish mutual awareness and intent sharing within the shared workspace between the human worker and robot [26]. A previous work developed a predictive model for agentic AI-empowered HRC that accurately forecasts a worker’s 3D motion in the context of the robot’s presence [27]. Importantly, this model moves beyond isolated human motion prediction by predicting how a human would move given the robot’s state. This proactive anticipation is essential to enable the robot as an intelligent and adaptive contributor in agentic AI-empowered HRC.

### 2.3. Knowledge Gap

Despite the theoretical promise of the agentic AI approach, agentic AI-empowered HRC systems still suffer from a fundamental lack of transparent and effortless communication modalities. This shortcoming means that existing AI-integrated HRC systems have a hard time interpreting users’ cues (mostly explicit) accurately and quickly, while autonomous robots struggle to convey their reasoning and intention to humans in a clear and low-effort manner. Previous studies have demonstrated that providing too much explanation about robot reasoning can contrarily lead to increased cognitive load. Construction workers already operate under significant cognitive demands due to the complexity of their tasks. Consequently, any additional load introduced by robotic integration could exacerbate the mental fatigue on the workforce.

While advanced agentic AI algorithms can autonomously reason and plan [23], a major gap remains in developing robust and scalable frameworks that effectively couple this intelligence with hardware systems. Deploying high-level AI reasoning in real-world settings requires the underlying hardware to operate reliably and consistently, which is particularly challenging in dynamic construction sites. Therefore, the current challenge is not in AI model capability, but in the lack of engineering methodologies for fusing ambiguous data from multimodal sources into the agent’s decision-making loop. There is a pressing need to address this gap by enhancing the system’s field robustness to ensure the reliable implementation of agentic-empowered AI in robotic systems.

To address these gaps, we propose a multimodal agentic AI framework that integrates NUIs via implicit cues (e.g., gaze and speech) into agentic AI for intuitive HRC. This system enables robots to reason, communicate, and translate multimodal human input into low-level robot control sequences, enhancing transparency and effortless interaction in HRC. It enhances the robustness of the LLM-enabled interfaces and allows for more flexible and efficient interactions between humans and robot hardware in teamwork.

## 3. Methodology

**Figure 2:**
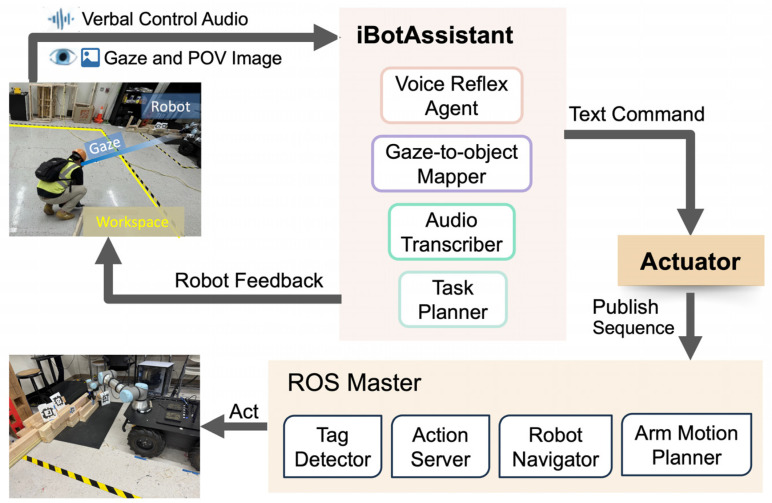
The framework of this study.

Our architecture creates an agentic AI workflow, iBotAssistant, for improved communication and efficient physical HRC in wood assembly task, utilizing natural user interfaces to derive automated robot control. It aims to assist human operators by reasoning their high-level verbal requirements (e.g., “Get me a lumber”) with the conjunction of implicit cues (gaze intention). This combination of explicit and implicit cues conveys explicit information from the person to the robot, while the person and the robot work together to complete a task. An overview of our system framework is shown in Figure 2. This framework includes three components: wearable devices for data collection, an agentic AI framework for LLM inference, and system integration for robot execution.

### 3.1. Agentic AI Framework for Intuitive HRC

In the context of HRC, human operators are tasked with assembly tasks while asking the robot for help verbally (e.g., “Get me a lumber”). The robot could identify the target using the human’s gaze data, plan its control sequences, and provide feedback for communication (e.g., “Which lumber are you referring to?”). As shown in Figure 2, the worker and the robot operate in a shared workspace, retrieving materials from a single storage area. First, a worker speaks to a robot through the wake word functionality, initializing interaction via “hey robot”. It starts to collect gaze-tracking data, a first-person point of view (POV) image, and records the worker’s audio. The data are collected and transmitted from the client laptop to a server.

Next, several AI agents are coordinated to process multiple types of information for accurate verbal interpreting (from language to robot actions) on a server. The image and gaze data are used to identify which object is the object of interest by the gaze-to-object mapper. Considering the low latency requirements in human–robot interactions, the time-consuming computer vision module was disabled in this study. Apriltags were applied in this case due to limited computing resources. Multiple tags were used to mimic a bunch of items sharing the same appearance scattered on a real construction site. An audio transcriber transcribes the recorded audio from the worker. The transcript and the target object (i.e., tag IDs) are sent to the task planner for the robot’s task reasoning and planning. Besides subtask generation, this planner also provides feedback to workers with the robot’s confirmation or questions. This feedback could reduce the worker’ uncertainty about robot movement and increase mutual communication between robots and workers.

Finally, the subtasks are fed to an actuator where the text in natural language is translated into robot control sequences published to the ROS master running on the robot. According to the different ROS nodes running on the robot, the actuator publishes control sequences as topic messages in corresponding formats. On the robot, tag detection is monitored by the tag detector to locate the target object. The action server is run to execute goal-oriented tasks. Built on an established ROS package, husky_navigation [28], a robot navigator package was developed. This is responsible for receiving tag IDs and navigating robot autonomously and collision-free in the workspace. An existing arm motion planner, husky_ur_manipulation, was applied to receive execution goals and plan trajectories for the arm [28]. The loop is defined in Algorithm 1.

**Algorithm 1:** **Multimodal System Execution Loop**

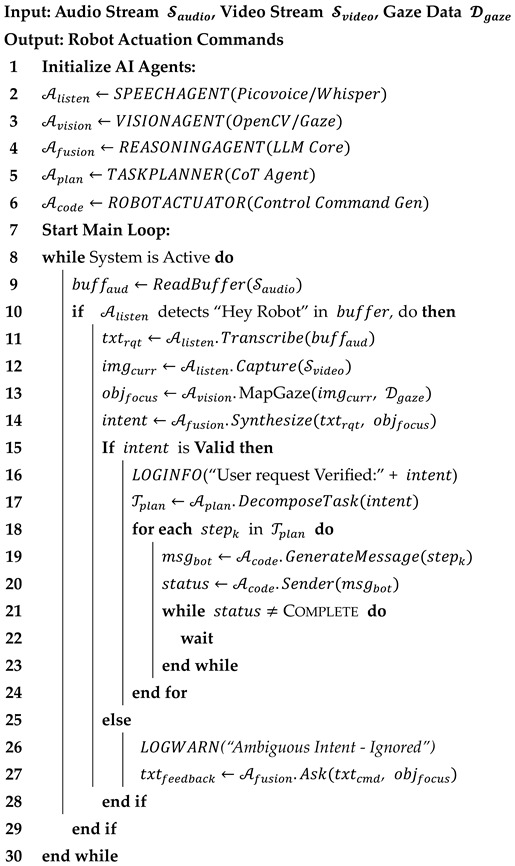



### 3.2. AI Agents and Tools

#### 3.2.1. Data Collection

Implicit and explicit cues are used as natural user interfaces in human robot interaction. Verbal communication is perceived one of the most efficient communication modalities in collaboration [29]. Users’ audio is captured when interacting with the robot. Gaze data are usually used to analyze human intention in object interactions. Specifically, it is applied in object manipulation tasks to inform the interested object when estimating human behaviors [30]. In such a scenario where a bundle of similar-appearing items are presented on the same site, gaze data are efficient to reveal human intentions precisely and are a compliment to eliminate the potential ambiguity of human language. Thus, gaze tracking was implemented and integrated into the language processing for robots to understand human intentions accurately. This method filtered out targets from various similar-looking objects (e.g., bunch of wood pieces). To trigger the interaction, the audio_trigger() agent continuously listens to the wake word through picovoice.ai [31]. Once it is detected, the gaze_extractor() agent initiates audio recording and gaze extraction to collect the user’s audio, gaze point, and image data. These data are sent by file_sender() to the assistant server.

#### 3.2.2. LLM Inference

**Figure 3:**
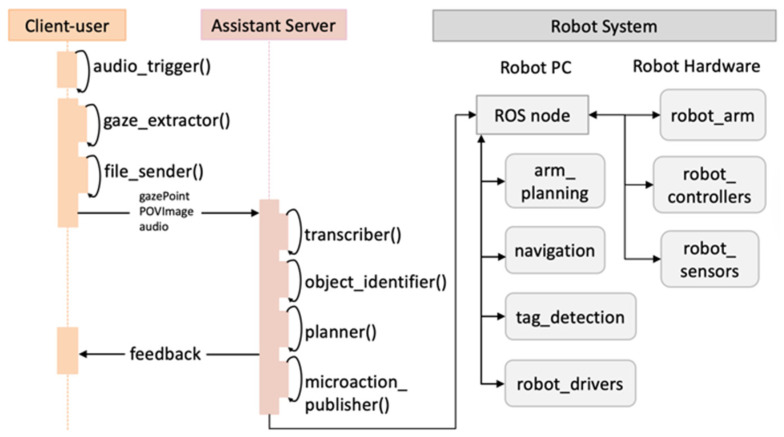
System communication dataflow.

AI agents have been used in social interactions and robot systems for reasoning tasks, translation, etc. [32]. An agentic AI workflow uses these AI agents to complete a complex task given by a person in a high-level description. This study leveraged several AI agents to form an agentic AI workflow to assist humans for intuitive HRC. Building on previous work, this paper details how an agentic AI framework can be implemented in real-world HRC with natural user interfaces (i.e., verbal and gaze) and how users evaluate it [23,33]. A semantic fusion mechanism was applied via context injection to process gaze, audio, and image, allowing the LLM’s reasoning in physical workspace and in dynamic context. The data flow among the client, server, and robot hardware is presented in Figure 3 to demonstrate our agentic AI workflow in HRC. As shown in Figure 3, three components, including user, assistant server, and robot system, form two client–server communications.

For multimodal processing, the assistant server serves as the main server and user laptop is the client. The server receives three types of data from the user client including gaze point, audio file, and the POV image. On the server, the transcriber() agent is used to transcribe the user’s audio to text as input for the planner() agent. Another input for planner() is generated by object_identifyer(), which identifies the robot’s interactive object specifically via gaze and image. The planner() agent returns planned subtasks for robot planning and provides feedback to humans for transparent communication. For robot execution, robot pc serves as a ROS server, and the assistant server is the client for the ROS master. The microaction_publisher() agent translates the robot’s subtasks generated from multimodal processing to robot executable orders. Then, it publishes control sequences one-by-one through topics to several ROS nodes. The ROS master continuously listens to those topics, and executes the orders on robot hardware including robot navigation and arm manipulation.

Another agent, transcriber(), uses OpenAI Whisper to transcribe from speech to text and output text. Apriltag and OpenCV libraries are applied in object_identifier() tool to detect and track tags for localizing robot interactive objects and locations. It also defines the target object by identifying the tag with the smallest distance between gaze point and the tag. The transcribed text and identified object represented by a tag are sent to the planner() agent. To ensure data privacy and reduce network latency, the framework is run locally instead of using a cloud-based LLM service like OpenAI’s API. This work used a tool, Shimmy, to implement the LLM [34]. This planner() agent is built on a light-weighted pre-trained LLM, the llama 3.2 quantized model [35]. It demonstrates proficiency in delivering instructions for task planning and interpreting human verbal input. This framework employs a hierarchical conflict resolution mechanism to prioritize verbal intent while utilizing gaze for spatial grounding. When gaze data are absent or fails, the system stops its execution to trigger a clarification request (robot asks back).

The output of this agent is regulated to include a verb and an object for each subtask, limited to objects such as wood blocks (robot interactive object in our case). The model was designed to cover a range of scenarios and tasks (i.e., “deliver”, “pick-up”, “place”, and “navigate”) in a possible construction collaboration, enabling effective translation between humans’ high-level requests and robot low-level control. An ask mode is also initiated to provide feedback to humans for clarification if their verbal request is uninterpretable. With the verbs that describe the robot’s micro-actions, a developed microaction_publisher() program translates these actions to executable robot commands. For example, the subtask outputs “pick up lumber with tag_5”. The translated robot command is “rostopic pub -1/ik_action_command std_msgs/String ‘pick tag_5’”. Such control commands are published sequentially to topics corresponding to the ROS node that takes in the command. The ROS nodes enabled in this case are detailed in the next section.

### 3.3. ROS System for Robot Execution

The information between the iBotAssistant and the physical robot is transmitted by the ROS master. The ROS master is hosted on the robot pc, running several packages for robot control. The robot used in this study was a four-wheeled unmanned ground vehicle, Husky A200, equipped with a Universal Robot 6-degree-of-freedom arm, UR5e. Thus, husky_navigation and amcl_navigation were used for the robot to localize itself on a pre-created map. Apriltag_ros was applied for the robot to detect and track tags for navigation and manipulation. Husky_base was consistently run to publish and receive robot status and messages for connecting robot drivers. Husky_ur_bringup and husky_ur_moveit_config were initiated to enable the robot arm for command receiving and motion planning. Despite the existing packages, two ROS packages were created for this system: tag_navigator enables the robot to navigate via Apriltags, and Ur_pick_place_ik integrates UR5e and its gripper to reach a location defined by an Apriltag through inverse kinematics.

## 4. Implementation

The proposed framework was implemented and demonstrated with a collaborative robot (a mobile robot with a manipulator and sensors). Seven participants were recruited to interact with this iBotAssistant-integrated robot in a timber assembly task to identify the primary usability for proof-of-concept implementation [36]. All participants had no to little experience with robots and shared age ranges from 25 to 34. Most participants had no construction work experience, but one participant held 6–9 years of work experience. The system integrates an eye tracker, a laptop running as a user-client, a workstation running as an assistant server, and a collaborative robot for physical interaction. An experimental task was designed and performed to apply our framework to a physical robot for real-world implementation. To evaluate the effectiveness of multimodal interaction, we compared the results with those of a baseline, verbal-only method (eye-tracker disabled). During the experiments, participants were asked to complete NASA TLX surveys [37] to collect evaluations of workload and conduct interviews about their perspectives on this system. A qualitative analysis was conducted to obtain practical findings of this implementation.

### 4.1. Experiment Setup

#### 4.1.1. Task Design

**Figure 4:**
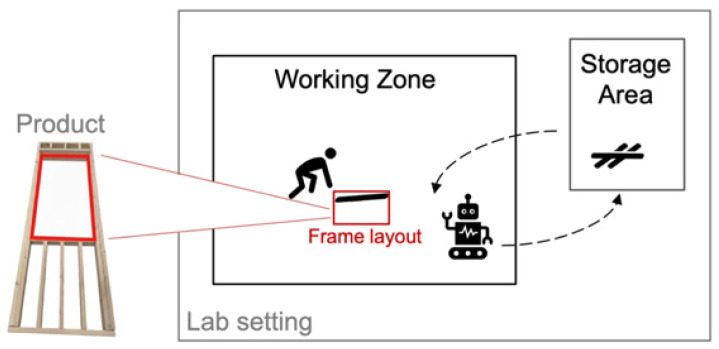
Task design and lab setup visualization. Red lines are the final product of the experiment, which was a window opening on framed wall.

The developed system was implemented in a construction timber assembly scenario. The collaborative robot assisted a human operator in framing window openings. A window frame in a lab setting was selected to limit the influence of external variables such as fatigue and environmental uncertainties. This window opening was taken from a standard 2 × 4 timber-framed wall for the assembly task, following the International Residential Code 2018 IRC602 [38]. The window size was set to 27-3/4 inches in width and 41-3/4 inches in height to reflect a two-pane side-hung casement. Next, 2 × 4 studs in 98 inches were cut to the designed lengths and used in this assembly task. As shown in Figure 4, the lab space had two zones—a workspace and a material storage zone—to reflect the settings on construction sites. The robot and the human operator could move between the two zones and complete the window frame in the shared workspace.

As shown in Table 1, the task is allocated based on the skill requirements and capabilities of different roles. The robot is tasked with delivering materials and holding a wood piece for the human, while the human worker is required to lay out the timbers on the floor and nail pieces for connection. The experiment was designed to follow the process of a timber assembly on a real construction site including the frame layout on the floor, parts delivery and placement, and nailing. To map subtasks to robot actions, the robot’s micro actions were defined in this task. In robot motion planning and control, the pick-and-place task has several steps, including motion planning, grabbing and holding, moving the object to a designated place, and placing it [39,40]. To link the subtasks to robot micro actions, the robot’s micro actions were identified based on functionalities such as “pick” and “release gripper”. Following a task allocation analytic network process, this assembly task was decomposed into functional actions for two roles (i.e., robot as helper and human as main carpenter).

#### 4.1.2. Experiment Process

The hardware configured in this study included a user’s PC, a workstation for the assistant server, an eye tracker, and a mobile robot equipped with an arm and a gripper. The built-in microphone on the user’s Pc was used to listen to the wake word and collect the user’s audio. A Pupil Core [41] was applied to collect gaze data. This device has been widely applied in various studies, such as social psychology studies, to obtain reliable eye tracking data. In our case, it collected human gaze data and the POV images for LLM inference. The workstation operated on a Ubuntu 20.04.6 with an Intel Xeon Gold 6230R, 125 GB RAM, and a Quadro RTX 6000. The robot used in this study was an unmanned ground vehicle, Husky A200 from Clear Path, equipped with a UR5e robotic arm from Universal Robot and a two-finger gripper from Robotiq. The robot hardware was configured with both the onboard PC and the workstation running ROS noetic. The necessary ROS packages were installed and configured.

In this setting, the worker engages in timber assembly tasks in the designated zones with a collaborative robot, as shown in Figure 4. During the experiment, the worker communicates their needs to the robot (e.g., requesting a specific working part) while looking at the desired object (i.e., lumber). This target object is located in a pile of lumber that shares a similar appearance. With a given order of requesting lumber, the robot is capable of navigating to the storage zone, picking up the specified lumber, and navigating back to place it on the ground. The human is responsible for connecting those pieces using a nail gun. The robot is also capable of holding the lumber for the participant to nail if requested by the user. Responses are generated in natural language for the robot to communicate with humans if more information is needed in a request.

**Figure 5:**
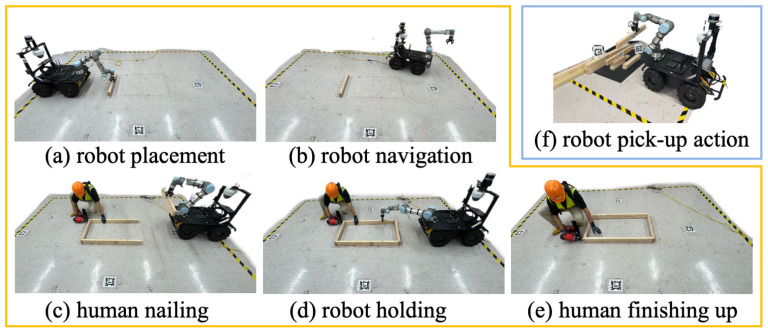
Experiment setup and procedures. Yellow box shows the working zone, and the blue box shows the storage area. (**a**,**b**,**f**) are the decomposed actions carried out by the robot to complete a lumber delivery request. (**c**–**e**) show a scene where the human and the robot are present in a shared space: (**c**) The robot delivers lumber while the human is nailing; (**d**) the human asks for assistance in holding; (**e**) the human is doing final checks and finishing up.

In the experiment, two conditions were established and evaluated (Figure 5). The first deployed the proposed framework, leveraging an agentic AI workflow for the robot to communicate and execute the worker’s requests in natural interactions (speech and gaze). The baseline operated without an eye-tracking device, relying solely on the verbal requests. The experiment sequence (gaze-guided followed by verbal-only) was fixed. Since the gaze-guided condition requires additional calibration and setup for eye tracker, keeping the conditions sequential allowed for a more stable technical environment. To facilitate a comparative analysis of the two approaches, the exact experiment process was conducted in both scenarios. Diverse requests were included to reflect real-life scenarios such as “get me the lumber” and “hold the lumber on the right side”.

### 4.2. Evaluation

To evaluate the effectiveness and overall quality of the experience from the human’s perspective, user experience metrics and a comparison of two scenarios (i.e., w/wo gaze) were used in this study. The proposed framework was compared to the baseline on task performance. This quantitative, objective evaluation measures the task completion of an HRC and the intervention rate in these two scenarios. To find out the practical insights of an agentic AI workflow in a real-world HRC application, a subjective evaluation was adopted, where we collected the humans’ feedback, opinions, and perceptions using interviews and surveys. A qualitative analysis was conducted to reveal valuable findings hidden behind the interview conversations.

#### 4.2.1. Task Performance

Two metrics were included in the task performance evaluation, following the evaluation criteria of human–robot interaction in other studies [42]. Task completion time was recorded to reflect the total time it took for the human and robot to finish a task collaboratively. Intervention counts were applied to measure how often the human had to step in to correct or assist the robot. The rate was calculated by measuring intervention counts over the total times the human gave a request to the robot. The scenario without an eye tracker was selected as the baseline for comparison.

#### 4.2.2. User Experience (UX) Metrics

**Figure 6:**
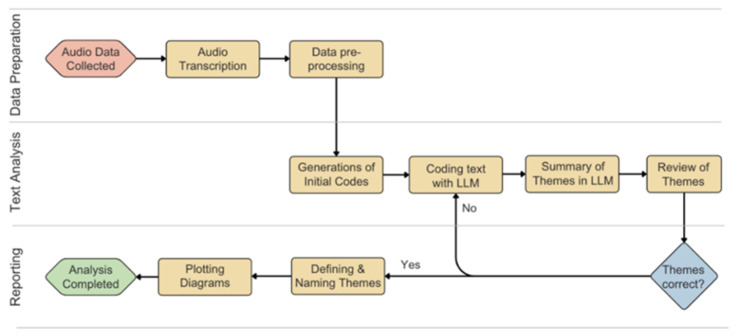
Qualitative analysis process flow.

As the mental workload is critically associated with workers’ performance in construction, measuring the workload of the proposed system is valuable [43]. NASA LTX was adopted to examine the mental and physical effort required to perform the task with the robot [37]. The humans’ workload was scored through a pairwise survey. The survey focused on six aspects including mental demand, physical demand, temporal demand, performance, effort, and frustration. Participants completed this survey twice, once for the system with the gaze system, and once for the system without. To evaluate the accessibility and provide insights into the users’ perceptions, a qualitative study was applied to the interview data via semantic analysis. We used a machine learning text-analysis tool, the Deep Computational Text Analyzer (DECOTA), to automatically code and derive themes for free-text data [44]. This is a fine-tuned LLM model developed by social and behavioral scientists to accelerate human coding in qualitative research. The data process flow for this study is shown in Figure 6.

## 5. Results and Discussion

### 5.1. Task Evaluation Results

The results and comparisons are listed in Table 2 and show that the proposed architecture can effectively translate the humans’ high-level requests through verbal and gaze into robot control commands for execution in an HRC task. The average intervention rate in the baseline doubled compared to that of the gaze-guided method, which indicates that the system needs participants to provide more information. The baseline system often asked to specify the object during the interaction because no target object data were obtained in the gaze-disabled scenario. On the other hand, the gaze-guided method also had an intervention rate because cases where the robot asked for a repeated request were counted as interventions. Notably, since ID#1 did not ask for robot assistance on material delivery, the intervention rate was roughly the same as the gaze-guided method.

Other participants had a higher intervention rate compared to the gaze-guided method. The average completion time of the baseline was lower than that of the gaze-guided method, even though the baseline had a longer interactive time (robot asking for clarification takes additional time). This could be because the experiment process and tasks remained the same for the two methods, resulting in higher familiarity with task procedures in the second experiment (i.e., baseline). Such a learning curve may contribute to the reduction in task completion time observed in subsequent experiments. In both scenarios, the timber frame was successfully assembled. Therefore, our agentic AI framework can effectively establish an intuitive verbal HRC, and with gaze enabled, the system outperformed the baseline in identifying the target from multiple similar or the same-looking objects.

### 5.2. UX Metrics Results

**Figure 7:**
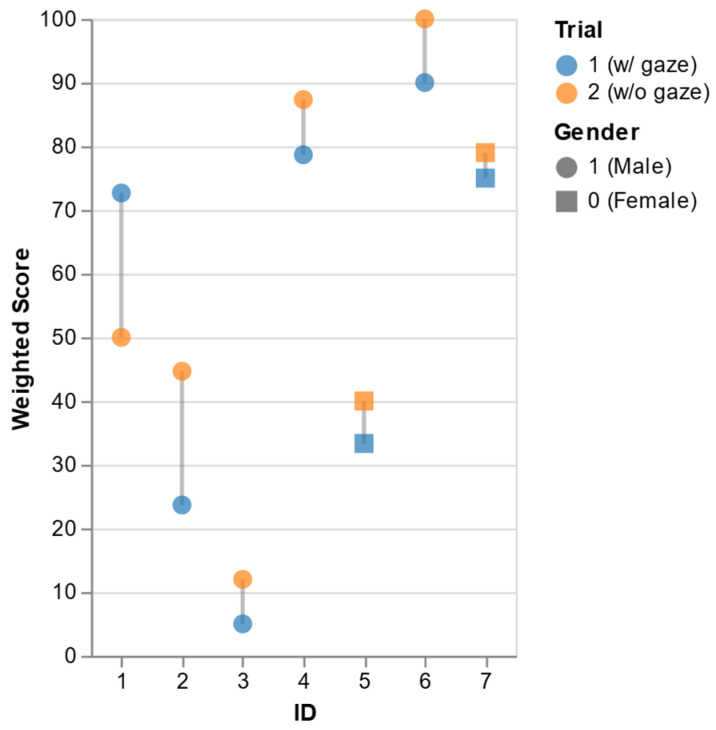
Change in weighted score per ID.

The results of NASA TLX are shown in Figure 7. Participants were instructed to evaluate the interactive system based exclusively on their primary experience, disregarding the high latency observed during the experiments. Six out of seven participants presented a higher workload score in the baseline scenario, but ID#1 lowered his score. This could be because ID#1 had fewer interactions with the robot—he did not correct the robot to deliver specified objects but transported all the lumber by himself. This indicates that the gaze-guided framework requires less effort to interact with in a practical HRC.

**Figure 8:**
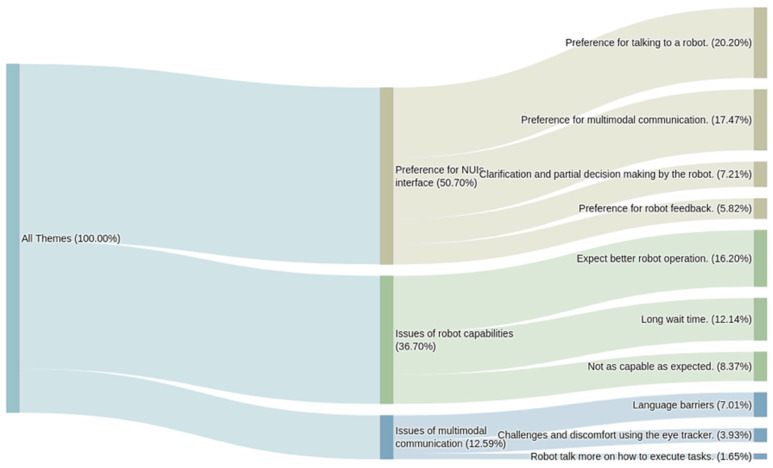
Sankey diagram of the question “How will you describe your experience of HRC?”.

The interviews were analyzed based on two questions. One question focused on the feedback of the interface: “How will you describe your experience of this HRC?”. The other question focused on ideas for developing the next-generation interface: “What features or changes will you make if you design the next version of the human–robot interface?” The results of the qualitative analysis are presented in Sankey diagrams to visualize the flow and proportion of themes. As shown in Figure 8, the majority of the participants mentioned the efficiency in human–robot interaction. Participants waited around ten seconds for the robot to process and execute due to the limited computing resources and data latency in transmission between the computers and the robot. The wait time was too long compared to the response time of 300 ms~500 ms in human–human interaction [45]. Communication robustness also stands out due to verbal recognition failure at some point. On the other hand, this framework establishes verbal communication between humans and robots to operate a real robot effortlessly. Interestingly, a proportion of the text points out the practical benefits of this system, suggesting its potential in improving work productivity.

**Figure 9:**
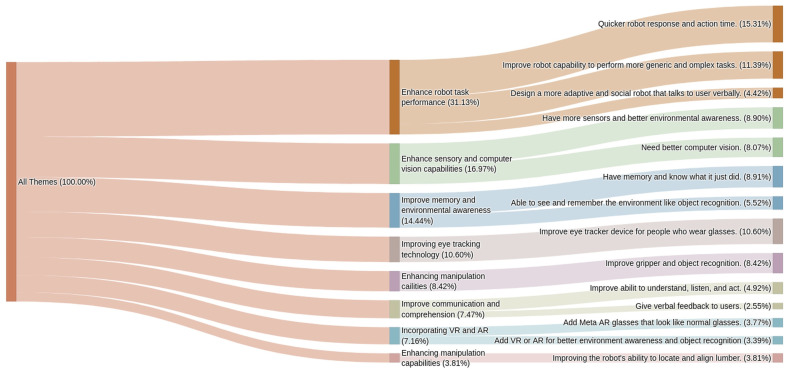
Sankey diagram of the question “What features or changes will you make if you design the next version of the human–robot interface?”.

Figure 9 demonstrates the density of different themes summarized by DECOTA. Robot capabilities and integration was one of the most mentioned themes. This is because when reinitialization of the robot hardware is required, different drivers initialize different devices, so it is possible that the robot’s gripper drops the object in the air as the arm has not been initialized yet. This integration is critical in avoiding such catastrophes on real construction sites. A major portion of the text also emphasizes enhancing the robot’s contextual understanding such as “add computer vision to have comprehensive environment understanding”.

### 5.3. Discussion

This study introduced and implemented a multimodal robot system architecture for real-world intuitive HRC. The evaluation results illustrate that the proposed agentic AI framework can establish intuitive and efficient HRC in real-world applications. It allows users to communicate and work with robots effortlessly through the intuitiveness of NUIs and the reasoning capability of LLMs. Our results demonstrate a relatively better interaction and higher user preference of the proposed method compared to the baseline, a pure LLM-method interface in an HRC assembly. On the other hand, almost all participants emphasized their favor of verbal interaction even though they experienced the advantage of gaze in conveying spatial information implicitly. This validates the significance of a language-centric interface design for HRC in complex environments like construction. This interesting finding reveals that extensive data integration is not always the best approach, but practical considerations need to be prioritized. When integrating different implicit and explicit cues, we need background knowledge down to the functional level, implementing groups, deploying environments, and thorough testing procedures. In our case, users who wore glasses daily significantly resisted wearing another glasses frame due to extra burden on their heads and faces. This may warrant the integration of eye tracking technologies into day-to-day wearable glasses.

In addition, our findings highlight critical requirements for the next generation of agentic AI-integrated HRC systems. The strong user expectation for extremely low latency underscores the need for optimized multi-agent communication and faster on-device processing and presents a long-standing challenge of real-time sensor fusion and data processing. Future research should focus on optimizing processing speeds through the integration of edge computing or specialized hardware like tensor processing units (TPUs). Furthermore, the demand for the memorization of prior task states emphasizes the necessity of integrating robust memory capabilities into the reasoning AI agent. This memory agent could not only perceive completed steps, but also be aware of the task blueprint to support multi-session tasks. Besides, robot capabilities also cover the ability to sense the environment and understand ambient dynamics. This can be concluded as the understanding of contextual information including partners (e.g., humans or robots), scene (e.g., working environment or objects), and changes (e.g., moving vehicles or obstacles). These findings pinpoint future research directions of optimizing the computational speed, contextual understanding, and logical state retention.

## 6. Conclusions and Limitations

This pilot study proposed an agentic AI framework that integrates multimodal NUIs (i.e., gaze and speech) to enhance HRC intuitiveness and transparency of communication between humans and robots. The system consists of three major components including data collection (from human eye-tracking, audio, and camera), the iBotAssistant, and robot system. It improves the efficiency and efficacy of interactions in HRC by conveying user intentions via implicit cues (i.e., gaze) and explicit cues (i.e., speech). To establish mutual, transparent communication, the robot provides feedback (i.e., confirmation/question) to users in natural language. In the real world, this framework was implemented as a proof-of-concept on a mobile robot with a manipulator, and seven participants were included in the timber assembly experiment. This study also compared the proposed method with the verbal-only method to evaluate its effectiveness. It shows that in scenarios where multiple objects have a similar appearance, the proposed method is more efficient than the verbal-only approach. Evaluation of human metrics indicates that users possessed a lower workload when using the proposed system. A qualitative analysis tool, DECOTA, was applied to find out valuable insights from the interview data. The results highlight the participants’ favor of the proposed interaction, especially verbal communication in HRC. The findings of these practical applications show that the robot’s integration and capabilities, such as integrating a memory system, lower processing duration, and more comprehensive contextual understanding, are expected to be improved. The results of the qualitative analysis provide user assessment on the feasibility, usability, and acceptability of humans for this practical agentic AI-framework implementation.

This study has some limitations. First, the agentic AI framework includes limited robot actions; it only executes tasks that contain actions defined in micro actions for robot control. More robot actions should be added to enrich the list for low-level control. Second, the small sample size of seven participants limits the generalizability of the quantitative findings. Future studies should include larger and more diverse participant pools to enable rigorous statistical analysis and validate the findings across different user groups and contexts. Third, tag detection was applied in the implementation, lacking a reflection of the real working environment. Due to limited computing resources, the object segmentation for robot perception was replaced by tag detection for tagged objects. Further research should integrate semantic object segmentation such as the segment anything model to enhance the system’s understanding of ambient including scenes, objects, passing vehicles, or workers. AI-context engineering can be integrated to further reinforce the contextual understanding of real construction sites for safety and reliability. In addition, a simplified timber structure was adopted in the experiment, which limits the assessment of the system’s feasibility. More interaction activities and HRC tasks should be included to evaluate the system’s effectiveness on various tasks. In the future, the agentic AI framework can be complemented for workflow professionals in different types of tasks by adding a logistic analysis agent, environment understanding agent, etc. Eventually, a multi-agent system is expected to autonomously and flexibly call different agents in demand to execute robot commands in HRC.

## Figures and Tables

**Table 1:** Task allocation analysis for timber framing task.

Role	Skill	Functional Action Allocation
Robot (helper)	Place, hold, scatter	1. Deliver and scatter the lumber 2. Place the lumber 3. Hold the lumber
Human	Nail, cut, measure	1. Measure and lay out the window on the floor 2. Mark and cut lumbers 3. Nail lumbers

**Table 2:** Experiment results and feedback.

Methods	#ID	Completion Time	Intervention Rate	Feedback Example
Gaze-guided (proposed)	1	11 m 40 s	10%	Confirmation
	2	10 m 28 s	20%	Confirmation
	3	12 m 08 s	10%	Confirmation
	4	13 m 30 s	30%	Confirmation
	5	13 m 20 s	20%	Confirmation
	6	9 m 02 s	20%	Confirmation
	7	14 m 57 s	20%	Confirmation
Mean		12 m 08 s	18.6%	
Verbal-only (baseline)	1	8 m 47 s	10%	None (didn’t ask robot to deliver a lumber)
	2	6 m 15 s	30%	“Which lumber are you referring to?”
	3	11 m 28 s	40%	“Please specify the lumber.”
	4	10 m 50 s	50%	“Didn’t get it. Please repeat.”
	5	8 m 44 s	40%	“Invalid plan. Please repeat your request.”
	6	8 m 03 s	40%	“Which lumber do you want me to grab?”
	7	12 m	50%	“Please specify which lumber you want.”
Mean		9 m 27 s	37.1%	

## Data Availability

The data presented in this study are available on request from the corresponding author due to privacy.

## Abbreviations

The following abbreviations are used in this manuscript:

AI	Artificial Intelligence
NUIs	Natural User Interfaces
HRC	Human–Robot Collaboration
LLMs	Large Language Models
NASA	The National Aeronautics and Space Administration
TLX	Task Load Index
BIC	Brain–Computer Interfaces
VR	Virtual Reality
POV	Point of View
UX	User Experience
DECOTA	Deep Computational Text Analyzer
